# Chemical Composition, Analgesic and Anti-Inflammatory Activity of *Pelargonium peltatum* Essential Oils from Eastern Cape, South Africa

**DOI:** 10.3390/molecules28145294

**Published:** 2023-07-08

**Authors:** Pamela Rungqu, Opeoluwa Oyedeji, Mavuto Gondwe, Adebola Oyedeji

**Affiliations:** 1 Department of Chemistry, Faculty of Science and Agriculture, University of Fort Hare, Alice 5700, South Africa; prungqu@gmail.com; 2 Department of Human Biology, Faculty of Health Science, Walter Sisulu University, Mthatha 5117, South Africa; mgondwe@wsu.ac.za; 3 Department of Chemistry, Faculty of Natural Sciences, Walter Sisulu University, Mthatha 5117, South Africa; aoyedeji@wsu.ac.za

**Keywords:** *Pelargonium peltatum*, chemical composition, acute toxicity, analgesic, anti-inflammatory

## Abstract

*Pelargonium* species are native to South Africa, and they have a long history in medicinal use. This study aimed to extract essential oils from different parts of *P. peltatum*, determine the chemical composition of the essential oils, and assess the essential oils’ biological potential as analgesic and anti-inflammatory agents. The essential oils were obtained by hydro-distilling different parts of *P. peltatum*, and the essential profile was determined by GC-FID and GC-MS. The analgesic activity of the essential oil was determined by using a tail immersion in hot water method in rats, whereas the anti-inflammatory activity of the essential oils was assessed according to right hind paw oedema induced by egg albumin; the three doses selected for each experiment were 100, 200, and 400 mg/kg. According to the GC-FID and GC-MS analysis, camphene (3.6–33.4%), α-terpineol (4.8–19.1%), α-thujone (1.5–15.6%), piperitone (0.9–12.2%), linalool (1.6–11.7%), myrcene (5.2–10.7%), germacrene D (3.7–10.4%), β-caryophyllene (1.2–9.5%), β-cadinene (3.4–6.7%), and β-bourbonene (4.2–6.2%) were some of the major compounds identified in the oil. *P. peltatum* essential oils demonstrated analgesic activity by increasing pain latency in hot water; furthermore, in an inflammation test, the essential oil reduced the egg-albumin-induced paw oedema in both the first and second phases. Therefore, the current findings suggest that *P. peltatum* essential oils have analgesic and anti-inflammatory properties.

## 1. Introduction

The *Pelargonium* genus belongs to the Geraniaceae family. The genus includes about 270 species, and the majority of these species are found in South Africa. For example, about 219 of the 270 species grow in southern Africa, while the remaining species are found in southeast and northeast Africa, Saint Helena, Asia, Madagascar, Tristan da Cunha, New Zealand, and Australia [1,2,3]. The *Pelargonium* genus (Geraniaceae) has a large number of species with scented leaves of various odours, ranging from pleasantly fruity or floral to rather oppressively balsamic [4]. *Pelargoniums* are an annual and herbaceous group of plants, shrubs, and subshrubs, all of which are deciduous and evergreen; however, most of the plants with scented leaves are succulent perennials in their native habitats. Some of the *Pelargonium* species grow upright, whereas others are trailing, and some have tuberous roots. *Pelargonium* stems are upright or decumbent (commonly with a woody base), soft-wooded or subsucculent (often viscid and aromatic), glandular, and variously hairy. *P. peltatum* (L.) L’Hérit. is commonly known as an ivy-leaved pelargonium or cascading geranium in English, kolsuring in Afrikaans, and umnewana or ityholo in isiXhosa. It is a climbing, semi-succulent, enduring plant, trailing through different trees and bushes in its environment [1,3,5]. *P. peltatum* has long, straggling shoots, which can grow up to a height of 2 m, and this plant is distinguishable because of its ivy-shaped leaves. The leaves are peltate, bluntly lobed, or five-angled; they are 2–7 cm in diameter and glabrous to hairy. Flowers are borne on two- to nine-flowered umbel-like inflorescences on long peduncles [6,7]. *P. peltatum* produces a bunch of flowers, which vary from mauve and pinkish mauve to pale pink and white. It flowers predominantly in September to December. *P. peltatum* is commonly found in sheltered places in seaside regions or succulent bush and dry rocky hillsides. This type of *Pelargonium* species is found in the Western Cape, Eastern Cape, Kwazulu-Natal, and Mpumalanga provinces of South Africa [1,3,8]. *P. peltatum* is also an invasive species, and it has been introduced to other countries and islands, such as Turkey [9], Bulgaria [10], France, Greece, Azores, the Canary Islands [11], Spain, New Zealand, the Galapagos Islands [12], and Ecuador [13]. *P. peltatum* leaves are traditionally used to treat sore throats, as the buds and the young leaves can be eaten and are said to be thirst quenching. The leaves can also be pounded and used as a disinfectant for scratches, wounds, grazes, and minor burns, and fresh leaves’ oils are used in treating toothaches and earaches. *P. peltatum* petals are astringent; they can be used in washing grease and for making a long-lasting grey-blue dye that can be used for painting or to dye wool or cloth [6,14]. This study aimed to extract essential oils from the different parts of *P. peltatum*, determine the chemical composition of the essential oils, and evaluate the biological potential of the essential oils as analgesic and anti-inflammatory agents. To the best of our knowledge, there are no reports on the chemical composition, analgesic activity, or anti-inflammatory activity of *P. peltatum* essential oils from the Eastern Cape, South Africa.

## 2. Results

### 2.1. Chemical Composition

The essential oils from *P. peltatum* leaf and twig exhibited a pale-yellow colour, and the odour of the oil was apple-like. The oil percentage yields were as follows: 1.6% for fresh leaf, 2.3% for dry leaf, 2.0% for fresh twig, and 2.5% for dry twig. The number of compounds identified in *P. peltatum* essential oils was fifty-three; Table 1 presents the identified compounds in *P. peltatum* essential oils, the percentage composition, and their Kovats indices. Seven compounds, accounting for 90.8% total oil composition in fresh leaf oil, were identified, while forty compounds accounted for 75.1% in dry leaf oil, thirty-five compounds accounted for 92.2% in fresh twig oil, and eleven compounds accounted for 87.7% in dry twig oil. Monoterpenes (43.5%) and oxygenated monoterpenes (43.0%) were the dominating chemical groups of compounds in the fresh leaf oil; the major compounds identified in the fresh leaf oil were camphene (33.4%), α-terpineol (19.1%), piperitone (12.2%), linalool (11.7%), myrcene (5.2%), β-pinene (4.9%), and β-caryophyllene (4.3%). The main compounds identified in the dry leaf oil were β-bourbonene (6.2%), phytone (5.0%), and α-terpineol (4.8%); furthermore, sesquiterpenes (13.8%), ketones (13.1%), monoterpenes (13.0%), and oxygenated monoterpenes (12.3%) were identified as the main chemical groups of compounds in the dry leaf oil. The fresh twig oil was dominated by sesquiterpenes (42.8%) and monoterpenes (30.8%), and the main representatives of the fresh twig oil were myrcene (10.7%), β-caryophyllene (9.5%), β-cubenene (5.6%), α-terpineol (5.4%), β-phellandrene (4.6%), and β-pinene (4.4%). In dry twig essential oil, oxygenated sesquiterpenes (34.9%) were identified as the most abundant chemical group of compounds, followed by sesquiterpenes (26.5%) and monoterpenes (16.4%) as the second and third most dominant groups of compounds in the oil; the principal compounds of the dry twig oil were α-thujone (15.6%), α-terpineol (13.1%), camphene (10.4%), germacrene D (10.4%), δ-cadinene (6.7%), linalool (6.2%), caryophyllene oxide (6.1%), β-pinene (6.0%), α-armophene (5.2%), and β-bourbonene (4.2%).

### 2.2. Acute Toxicity

The essential oils of *P. peltatum* (fresh leaf, dry leaf, fresh twig, and dry twig) were tested for acute toxicity in mice by oral administration. The acute toxicity results showed that the essential oils of *P. peltatum* caused no mortality in mice at the highest dosage of 5000 mg/kg (Table 2). The animals showed no signs of toxicity or change in general behaviour or other physical activities.

### 2.3. Analgesic Activity

#### 2.3.1. Effect of *P. peltatum* Fresh Leaf Essential Oil on Tail Immersion of Rats in Hot Water

**Figure 1:**
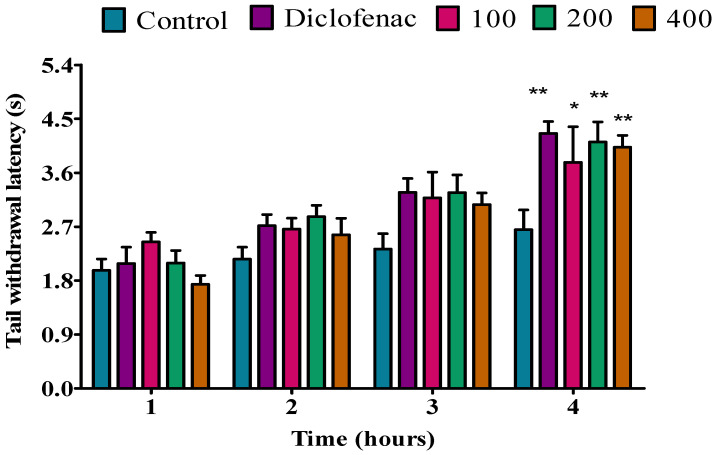
Effect of *P. peltatum* fresh leaf (P.P.F.L) essential oil on thermal pain induced in rats. Control, diclofenac, 100, 200, and 400 bars; represent negative control (tween 80 and distilled water (10 mg/kg)), diclofenac 100 mg/kg, 100, 200, and 400 mg/kg, respectively. * *p* < 0.05, ** *p* < 0.01 statistically significant compared to control.

**Figure 2:**
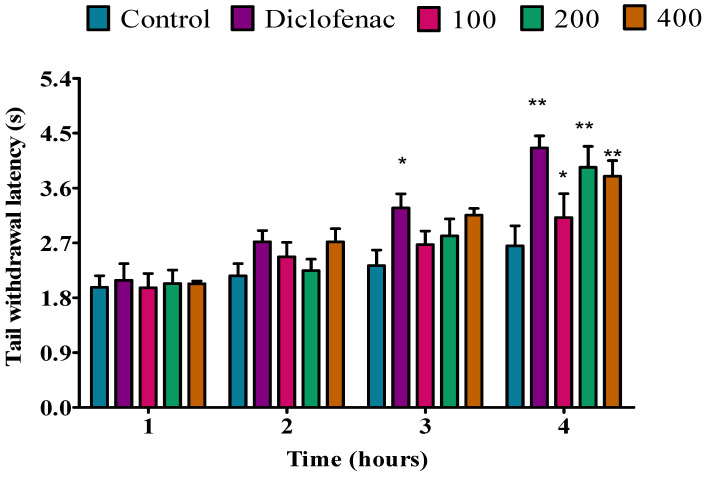
Effect of *P. peltatum* dry leaf (P.P.D.L) essential oil on thermal pain induced in rats. Control, diclofenac, 100, 200, and 400 bars; represent negative control (tween 80 and distilled water (10 mg/kg)), diclofenac (100 mg/kg), 100, 200, and 400 mg/kg, respectively. * *p* < 0.05, ** *p* < 0.01 statistically significant compared to control.

**Figure 3:**
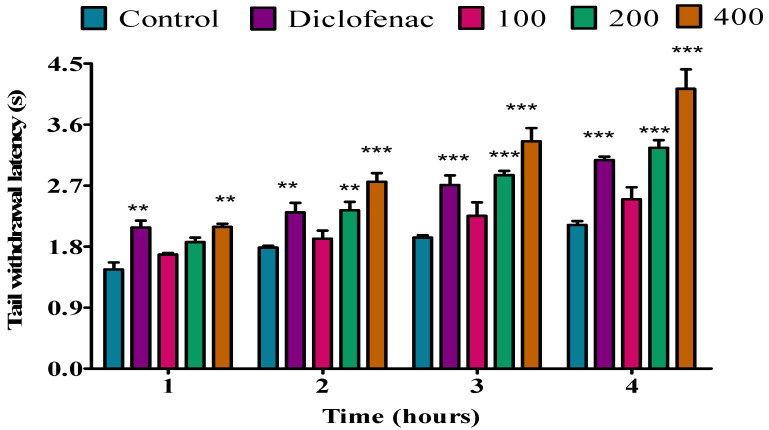
Effect of *P. peltatum* fresh twig (P.P.F.T) essential oil on thermal pain induced in rats. Control, diclofenac, 100, 200, and 400 bars; represent negative control (tween 80 and distilled water (10 mg/kg)), diclofenac (100 mg/kg), 100, 200, and 400 mg/kg, respectively. ** *p* < 0.01, *** *p* < 0.001 statistically significant compared to control.

**Figure 4:**
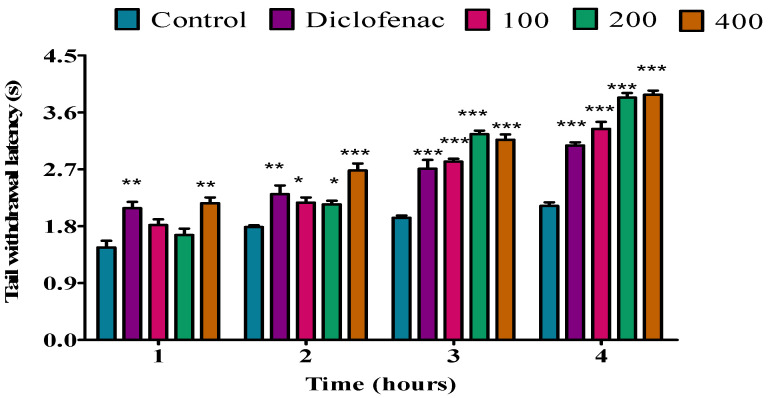
Effect of *P. peltatum* dry twig (P.P.D.T) essential oil on thermal pain induced in rats. Control, diclofenac, 100, 200, and 400 bars; represent negative control (tween 80 and distilled water (10 mg/kg)), diclofenac (100 mg/kg), 100, 200, and 400 mg/kg, respectively. * *p* < 0.05, ** *p* < 0.01, *** *p* < 0.001 statistically significant compared to control.

The analgesic activity of the *P. peltatum* essential oil of fresh leaf, dry leaf, fresh twig, and dry twig was evaluated by a tail immersion method in rats. The analgesic activity results are shown in Figure 1, Figure 2, Figure 3 and Figure 4. The fresh leaf essential oil of *P. peltatum* showed no analgesic effect at the first, second, or third hour at doses of 100, 200, or 400 mg/kg when compared to the control; no analgesic effect was observed for diclofenac. However, at the fourth hour, the *P. peltatum* fresh leaf essential oil showed a significant (*p* < 0.05) analgesic effect when compared to the control at a dose of 100 mg/kg. However, when compared to diclofenac, diclofenac showed a highly significant (*p* < 0.01) analgesic effect. Moreover, a significant (*p* < 0.01) analgesic effect was observed at doses of 200 and 400 mg/kg compared to the control, but similar results were observed when compared to diclofenac (Figure 1).

#### 2.3.2. Effect of *P. peltatum* Dry Leaf Essential Oil on Tail Immersion of Rats in Hot Water

In *P. peltatum* dry leaf essential oil, no analgesic effect was observed during the first, second, or third hour at doses of 100, 200, or 400 mg/kg. Diclofenac was inactive during the first and second hour; however, at the third hour, diclofenac exhibited a significant (*p* < 0.05) analgesic activity. At a dose of 100 mg/kg during the fourth hour, the dry leaf essential oil showed significant (*p* < 0.05) analgesic activity when compared to the control. Furthermore, at doses of 200 and 400 mg/kg, the dry leaf oil exhibited a significant (*p* < 0.01) analgesic effect when compared to the control. However, similar results (*p* < 0.01) were observed when the dry leaf essential oil was compared to diclofenac (Figure 2).

#### 2.3.3. Effect of *P. peltatum* Fresh Twig Essential Oil on Tail Immersion of Rats in Hot Water

The fresh twig essential oil was inactive at doses of 100 and 200 mg/kg during the first hour; however, at a dose of 400 mg/kg, the fresh twig essential oil showed a significant (*p* < 0.01) analgesic activity compared to the control. However, when compared to diclofenac, similar results were observed. No analgesic activity was observed for fresh twig essential oil at a dose of 100 mg/kg during the second hour, but at a dose of 200 mg/kg, the fresh twig essential oil exhibited a significant (*p* < 0.01) analgesic activity when compared to the control; similar results were observed when compared to diclofenac. At a dose of 400 mg/kg during the second hour, fresh twig essential oil showed a significant (*p* < 0.001) analgesic activity when compared to the control. In addition, the dose of 400 mg/kg showed a good analgesic effect when compared to diclofenac. At a dose of 100 mg/kg during the third and fourth hour, no analgesic activity was observed for the fresh twig essential oil; however, at doses of 200 and 400 mg/kg, the fresh twig essential oil showed significant (*p* < 0.001) analgesic activity when compared to the control. Similar results were observed when compared to diclofenac (Figure 3).

#### 2.3.4. Effect of *P. peltatum* Dry Twig Essential Oil on Tail Immersion of Rats in Hot Water

No analgesic activity was observed at doses of 100 or 200 mg/kg in dry twig essential oil during the first hour; however, at a dose of 400 mg/kg, the dry twig essential oil exhibited a significant (*p* < 0.01) analgesic effect compared to the control, and similar results were observed when compared to diclofenac. During the second hour, at doses of 100 and 200 mg/kg, the dry twig essential oil showed a significant (*p* < 0.05) analgesic effect when compared to the control, while diclofenac exhibited significant (*p* < 0.01) analgesic activity. Additionally, at a dose of 400 mg/kg, a good significant (*p* < 0.001) analgesic effect was observed for the dry twig essential oil. At the third and fourth hour, the dry twig essential oil showed a significant (*p* < 0.001) analgesic effect when compared to the control at doses of 100, 200, and 400 mg/kg; similar results were observed when compared to diclofenac (Figure 4).

### 2.4. Anti-Inflammatory Activity

#### 2.4.1. Effect of *P. peltatum* Fresh Leaf Essential Oil on Egg-Albumin-Induced Paw Oedema in Rats

**Figure 5:**
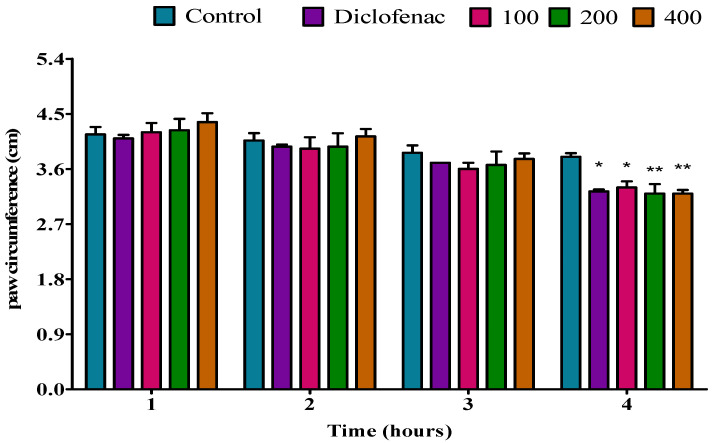
Effect of *P. peltatum* fresh leaf (P.P.F.L) essential oil on egg-albumin-induced paw oedema in rats. Control, diclofenac, 100, 200, and 400 bars; represent negative control (tween 80 and distilled water (10 mg/kg)), diclofenac (100 mg/kg), 100, 200, and 400 mg/kg, respectively. * *p* < 0.05, ** *p* < 0.01 statistically significant compared to control.

**Figure 6:**
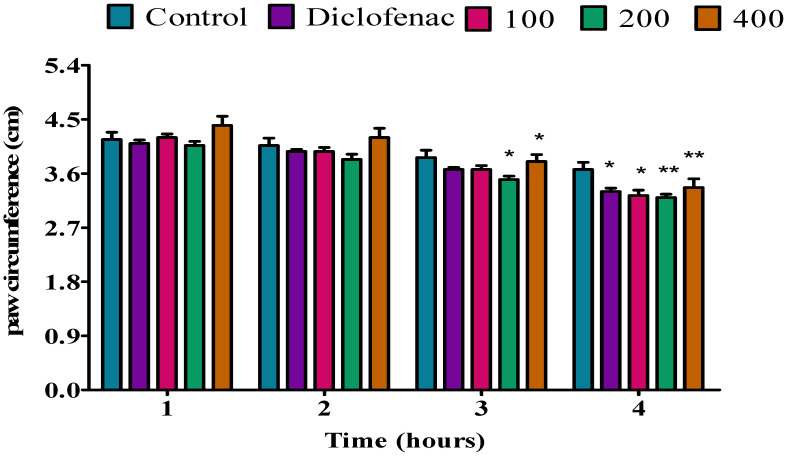
Effect of *P. peltatum* dry leaf (P.P.D.L) essential oil on egg-albumin-induced paw oedema in rats. Control, diclofenac, 100, 200, and 400 bars; represent negative control (tween 80 and distilled water (10 mg/kg)), diclofenac (100 mg/kg), 100, 200, and 400 mg/kg, respectively. * *p* < 0.05, ** *p* < 0.01 statistically significant compared to control.

**Figure 7:**
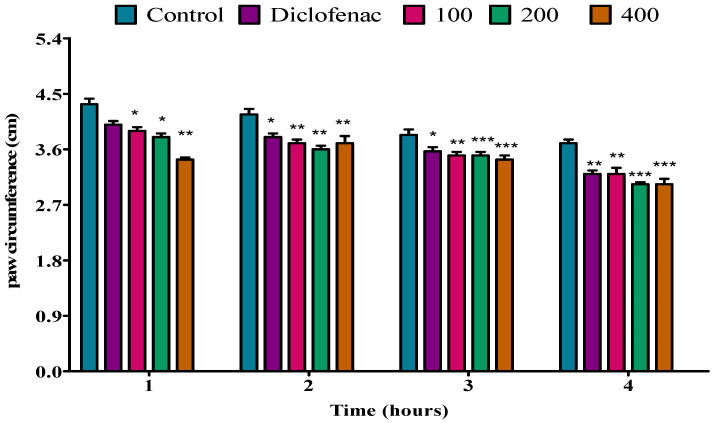
Effect of *P. peltatum* fresh twig (P.P.F.T) essential oil on egg-albumin-induced paw oedema in rats. Control, diclofenac, 100, 200, and 400 bars; represent negative control (tween 80 and distilled water (10 mg/kg)), diclofenac (100 mg/kg), 100, 200, and 400 mg/kg, respectively. * *p* < 0.05, ** *p* < 0.01, *** *p* < 0.001 statistically significant compared to control.

**Figure 8:**
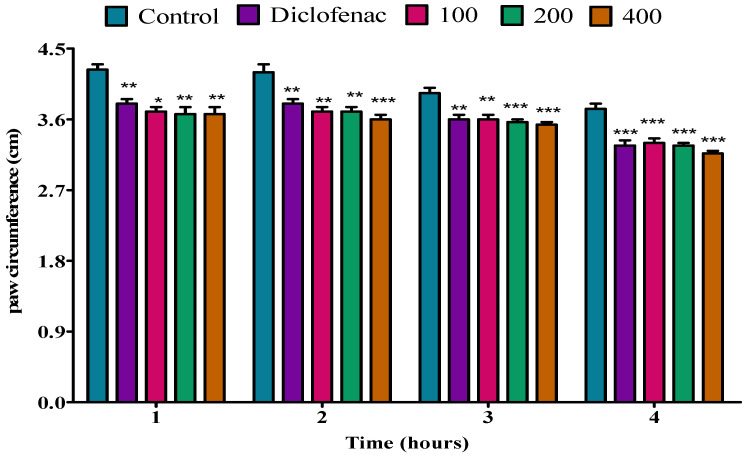
Effect of *P. peltatum* dry twig (P.P.D.T) essential oil on egg-albumin-induced paw oedema in rats. Control, diclofenac, 100, 200, and 400 bars; represent negative control (tween 80 and distilled water (10 mg/kg)), diclofenac (100 mg/kg), 100, 200, and 400 mg/kg, respectively. * *p* < 0.05, ** *p* < 0.01, *** *p* < 0.001 statistically significant compared to control.

Fresh egg-albumin-induced oedema was used to test the anti-inflammatory effect of *P. peltatum* essential oils of fresh leaf, dry leaf, fresh twig, and dry twig; the results are shown in Figure 5, Figure 6, Figure 7 and Figure 8. All three doses of 100, 200, and 400 mg/kg of *P. peltatum* fresh leaf essential oil and diclofenac exhibited no anti-inflammatory effect on egg-albumin-induced oedema in rats during the first, second, or third hour. However, during the fourth hour, the fresh leaf essential oil substantially (*p* < 0.05) reduced the paw oedema at a dose of 100 mg/kg compared to the control; similar results were observed when compared to diclofenac, and at doses of 200 and 400 mg/kg, the fresh leaf essential oil showed a significant (*p* < 0.01) inhibition of the paw oedema when compared to the control. However, diclofenac showed a low (*p* < 0.05) reduction of paw oedema when compared to 200 and 400 mg/kg doses of fresh leaf essential oil (Figure 5).

#### 2.4.2. Effect of *P. peltatum* Dry Leaf Essential Oil on Egg-Albumin-Induced Paw Oedema in Rats

No anti-inflammatory effect was observed for diclofenac or any of the doses of 100, 200, or 400 mg/kg of dry leaf essential oil during the first or second hour. Dry leaf essential oil showed a significant (*p* < 0.05) anti-inflammatory activity on egg-albumin-induced paw oedema at doses of 200 and 400 mg/kg during the third hour compared to the control; however, no anti-inflammatory activity was observed for diclofenac at the third hour. Moreover, the dry leaf essential oil exhibited significant (*p* < 0.05) anti-inflammatory activity at a dose of 100 mg/kg during the fourth hour compared to the control, and similar results were observed for diclofenac. Furthermore, at doses of 200 and 400 mg/kg, dry leaf essential oil showed significant (*p* < 0.01) anti-inflammatory activity compared to the control. However, diclofenac showed low (*p* < 0.05) anti-inflammatory activity relative to 200 and 400 mg/kg doses of dry leaf essential oil (Figure 6).

#### 2.4.3. Effect of *P. peltatum* Fresh Twig Essential Oil on Egg-Albumin-Induced Paw Oedema in Rats

The fresh twig essential oil significantly (*p* < 0.05) reduced the egg-albumin-induced paw oedema at doses of 100 and 200 mg/kg during the first hour compared to the control; at a dose of 400 mg/kg, the fresh twig essential oil showed significant (*p* < 0.01) anti-inflammatory activity compared to the control. No anti-inflammatory effect was observed for diclofenac at the first hour. During the second hour, the fresh twig essential oil showed a substantial (*p* < 0.01) inhibition of the paw oedema at doses of 100, 200, and 400 mg/kg compared to the control, while diclofenac exhibited a significant (*p* < 0.05) inhibition of the paw oedema, which was lower than that of the fresh twig essential oil doses. At the third hour, the fresh twig essential oil significantly (*p* < 0.01) reduced the paw oedema at a dose of 100 mg/kg compared to the control. In addition, at doses of 200 and 400 mg/kg, the fresh twig essential oil showed a substantial (*p* < 0.001) reduction of the paw oedema compared to the control, but diclofenac showed lower (*p* < 0.05) anti-inflammatory activity. During the fourth hour, at a dose of 100 mg/kg, the fresh twig essential oil exhibited a substantial (*p* < 0.01) inhibition of the paw oedema induced by egg albumin compared to the control; similar results were observed when compared to diclofenac. Moreover, at doses of 200 and 400 mg/kg, the fresh twig essential oil significantly (*p* < 0.001) reduced the paw oedema compared to the control; however, diclofenac showed a lower significant (*p* < 0.01) anti-inflammatory effect (Figure 7).

#### 2.4.4. Effect of *P. peltatum* Dry Twig Essential Oil on Egg-Albumin-Induced Paw Oedema in Rats

Dry twig essential oil significantly (*p* < 0.05) reduced the paw oedema at a dose of 100 mg/kg during the first hour compared to the control, but diclofenac exhibited a higher significant (*p* < 0.01) anti-inflammatory effect. Furthermore, at doses of 200 and 400 mg/kg, the dry twig essential oil showed a significant (*p* < 0.01) inhibition of the paw oedema relative to the control; similar results were observed when compared to diclofenac. At doses of 100 and 200 mg/kg, dry twig essential oil significantly (*p* < 0.01) reduced the paw oedema at the second hour compared to the control; similar results were observed when compared to diclofenac. However, at a dose of 400 mg/kg, dry twig essential oil caused a substantial (*p* < 0.001) inhibition of the paw oedema in rats relative to the control; diclofenac exhibited lower substantial (*p* < 0.01) anti-inflammatory activity relative to the 400 mg/kg dose. During the third hour, at a dose of 100 mg/kg, dry twig essential oil showed a significant (*p* < 0.01) inhibition of the paw oedema compared to the control; when compared to diclofenac, similar results were observed. Additionally, at doses of 200 and 400 mg/kg, the dry twig essential oil caused a significant (*p* < 0.001) reduction in the paw oedema caused by egg albumin compared to the control; when compared to diclofenac, a lower significant (*p* < 0.01) anti-inflammatory effect was observed at the third hour. At doses of 100, 200, and 400 mg/kg, during the fourth hour, the dry twig essential oil significantly (*p* < 0.001) reduced the paw oedema compared to the control; similar results were observed when compared to diclofenac (Figure 8).

## 3. Discussion

### 3.1. Chemical Composition of P. peltatum Essential Oils

This study was able to determine the chemical composition, acute toxicity, analgesic and anti-inflammatory activity of fresh leaf, dry leaf, fresh twig, and dry twig of *P. peltatum* essential oils. *P. peltatum* growing from the Eastern Cape in South Africa has not been subjected to any chemical composition, analgesic activity, or anti-inflammatory activity investigation. Therefore, this will be the first report on the chemical composition, analgesic and anti-inflammatory activity of *P. peltatum* essential oils from the Eastern Cape in South Africa. According to our results, *P. peltatum* essential oils had camphene (33.4%), α-terpineol (19.1%), piperitone (12.2%), linalool (11.7%), myrcene (5.2%), β-pinene (4.9%), and β-caryophyllene (4.3%) as the principal compounds in fresh leaf oil. Meanwhile, dry leaf oil was dominated by β-bourbonene (6.2%), phytone (5.2%), and α-terpineol (4.8%). Fresh twig oil had myrcene (10.7%), β-caryophyllene (9.5%), β-cubenene (5.6%), α-caryophyllene (5.5%), β-phellandrene (4.6%), and β-pinene (4.4%) as the predominant compounds of the oil. Meanwhile, in dry twig oil, α-thujone (15.6%), α-terpineol (13.1%), camphene (10.4%), germacrene D (10.4%), β-cadinene (6.7%), linalool (6.2%), caryophyllene oxide (6.1%), β-pinene (6.0%), α-armophene (5.2%), and β-bourbonene (4.2%) were identified as major compounds. There is not much data on the chemical composition of *P. peltatum* essential oils. However, the chemical composition of *P. peltatum* essential oils has been studied in Egypt, and those results differed significantly from those reported in this study. For instance, according to Nossier 2019 [18] (p.i), five compounds were identified in the Egyptian *P. peltatum* essential oils: β-myrcene (44.86%), β-pinene (35.89%), α-pinene (10.40%), p-mentha-1(7),8-diene (5.14%), and camphene (3.17%). The dominant compounds identified in this study were camphene (3.6–33.4%), α-terpineol (4.8–19.1%), α-thujone (1.5–15.6%), piperitone (0.9–12.2%), linalool (1.6–11.7%), β-myrcene (5.2–10.7%), germacrene d (3.7–10.4%), β-caryophyllene (1.2–9.5%), β-cadinene (3.4–6.7%), β-bourbonene (4.2–6.2%), caryophyllene oxide (1.9–6.1%), β-pinene (1.4–6.0%), β-cubenene (5.6%), α-caryophyllene (0.8–5.5%), α-armophene (1.2–5.2%), phytone (0.4–5.0%), and β-phellandrene (4.6%).

Upon comparing the results of this study with those from the Egyptian study, we found that the *P. peltatum* essential oils from Egypt had a high content of β-myrcene (44.86%) and β-pinene (35.89%). β-myrcene and β-pinene were also identified in this study, but they were present in insignificant amounts (of 5.2–10.7% and 1.4–6.0%, respectively). Moreover, α-pinene (10.40%) and p-mentha-1(7),8-diene (5.14%), which were identified as the major compounds of the Egyptian *P. peltatum* essential oil, were not identified in this study. Furthermore, camphene was present in small amounts in the Egyptian *P. peltatum* essential oil, while in this study, camphene was present at a high concentration. Additionally, some of the major compounds identified in this study, such as α-terpineol (4.8–19.1%), α-thujone (1.5–15.6%), piperitone (0.9–12.2%), linalool (1.6–11.7%), germacrene D (3.7–10.4%), β-caryophyllene (1.2–9.5%), β-cadinene (3.4–6.7%), β-bourbonene (4.2–6.2%), caryophyllene oxide (1.9–6.1%), β-cubenene (5.6%), α-caryophyllene (0.8–5.5%), α-armophene (1.2–5.2%), phytone (0.4–5.0%), and β-phellandrene (4.6%), were not present in the Egyptian *P. peltatum* essential oil. The chemical variability found in the chemical composition of the essential oils of *P. peltatum* from this study and the study from Egypt might be due to the part of the plant used, the method of extraction, the geographical location, and the climatic conditions [19,20].

### 3.2. Acute Toxicity and Analgesic Activity of P. peltatum Essential Oils

The toxicity tests of *P. peltatum* (fresh leaf, dry leaf, fresh twig, and dry twig) essential oils caused no mortality in mice at the highest dosage of 5000 mg/kg (Table 2). The animals showed no signs of toxicity or change in general behaviour or other physical activities. Therefore, this explains the plants’ widespread use in traditional medicine. The analgesic activity of *P. peltatum* (fresh leaf, dry leaf, fresh twig, and dry twig) essential oil was analysed using a tail immersion method. While the anti-inflammatory activity of the essential oils was determined by an egg-albumin-induced paw oedema, the activities were determined at doses of 100, 200, and 400 mg/kg. The reference drug that was used was diclofenac. The analgesic effect of *P. peltatum* fresh and dry leaf essential oil increased with the passage of time, and a good analgesic effect was achieved at the fourth hour at doses of 200 and 400 mg/kg. Meanwhile, the fresh and dry twig essential oils showed maximum activity at doses of 200 and 400 mg/kg from the first to the fourth hour, with an increase in pain reaction time. The analgesic effect of *P. peltatum* might be associated with the presence of β-pinene, β-caryophyllene, germacrene D, linalool, camphene, caryophyllene oxide, α-terpineol, and myrcene, which were identified as some of the principal compounds found in *P. peltatum* essential oils.

Previous studies have revealed that animals that were treated with *Ocimum gratissimum* oil and myrcene showed increased latency of paw withdrawal from the hot plate; furthermore, the animals showed a reduction in licking times of the paw in both the first and second phases [21]. Lavender essential oil has been reported to possess analgesic activity; linalool is one of the dominating compounds in the oil [22]. According to Tashiro et al. [23] (pp. 1–11), linalool odor exposure significantly decreased pain response in the first and the second phase of a formalin test in mice, whereas in a hot plate test, linalool increased the latency of hind paw withdrawal. In a study performed by Pinho et al. [24] (pp. 681–685), the essential oil of *Ocimum micranthum* showed that the analgesic activity of the essential oil might be due to the presence of β-caryophyllene. Moreover, Klauke et al. [25] (pp. 608–620) investigated the analgesic effects of β-caryophyllene in animal models of neuropathic and inflammatory pain, and the results indicated that β-caryophyllene may be highly effective in treating long lasting, weakening pain states. β-pinene, which was one of the major compounds identified in the essential oil of *Eucalyptus camaldulensis*, exerted supraspinal antinociceptive actions in rats [26]. Furthermore, Lima et al. [27] (pp. 274–282) studied the antinociceptive activity of the essential oil of *Piper alyreamum* leaves using the formalin test; β-pinene, camphene, and caryophyllene oxide were identified as some of the major compounds of the oil, and the results showed that the essential oil significantly inhibited both the inflammatory and neurogenic phases of formalin-induced licking. Furthermore, in the work conducted by Shah et al. [28] (p. 244), the GC-MS analysis showed the presence of myrcene, germacrene D, and β-caryophyllene as some of the major compounds of *Teucrium stocksianum* essential oil. When the oil was tested for antinociceptive activity using the acetic-acid-induced writhing test, the oil decreased the amount of writhing. The analgesic activity of *P. peltatum* might be due to the inhibition of prostaglandin pathways or through a peripheral pain mechanism [29].

### 3.3. Anti-Inflammatory Activity of P. peltatum Essential Oils

The anti-inflammatory activity of *P. peltatum* essential oils from fresh leaf, dry leaf, fresh twig, and dry twig was assessed by employing an in vivo model of egg-albumin-induced paw oedema. The presence of oedema is one of the primary signs of inflammation. Oedema formation is due to the synergistic action of inflammatory mediators, such as histamine, serotonin, and bradykinin, at the site of a local inflammatory insult, which leads to an increase in vascular permeability and blood flow [30]. Oedema formation due to egg albumin in the rat paw is biphasic, and the early phase of oedema, which occurs immediately after the phlogistic agent injection and lasts up to 2 h, is probably due to the release of histamine and serotonin, while the late phase, which occurs from 3 h to 5 h after the phlogistic agent injection, is due to bradykinin, protease, prostaglandins, and lysosome [31].

No anti-inflammatory effect was observed for diclofenac or *P. peltatum* fresh leaf essential oil doses of 100, 200, or 400 mg/kg from the first to the third hour; however, during the fourth hour, the fresh leaf essential oil significantly (*p* < 0.05–0.01) reduced the paw oedema in all doses of 100, 200, and 400 mg/kg. Meanwhile, diclofenac showed low inhibition (*p* < 0.05) during the fourth hour when compared to the fresh leaf oil. Diclofenac and dry leaf essential oil were insignificant from the first to the second hour, but at the third and fourth hour, the dry leaf essential oil significantly (*p* < 0.05–0.01) inhibited the paw oedema after egg albumin injection at 100, 200, and 400 mg/kg doses. Meanwhile, diclofenac showed low significant (*p* < 0.05) anti-inflammatory activity. The essential oil from the fresh twig effectively inhibited (*p* < 0.05–0.01) egg-albumin-induced paw oedema at doses of 100, 200, and 400 mg/kg during the first hour, whereas diclofenac showed no inhibitory effect. At the second hour, the fresh twig essential oil significantly (*p* < 0.01–0.001) reduced the paw oedema at doses of 100, 200, and 400 mg/kg, while diclofenac poorly inhibited (*p* < 0.05) the egg-albumin-induced oedema. During the third and fourth hour, the fresh twig essential oil showed a reduction (*p* < 0.01–0.001) of paw oedema at doses of 100, 200, and 400 mg/kg, whilst diclofenac exhibited an inhibitory (*p* < 0.01) effect compared to the fresh twig essential oil at doses of 200 and 400 mg/kg. The dry twig essential oil significantly (*p* < 0.05–0.01) inhibited the paw oedema in the first and second hours of inflammation induced by egg albumin at doses of 100, 200, and 400 mg/kg. Meanwhile, for diclofenac, results of anti-inflammatory activity similar to those of the 200 and 400 mg/kg doses were observed. During the third and fourth hour, the dry twig essential oil effectively inhibited (*p* < 0.01–0.001) the egg-albumin-induced paw oedema at doses of 100, 200, and 400 mg/kg; diclofenac significantly (*p* < 0.01) reduced the egg-albumin-induced oedema. The anti-inflammatory activity of the *P. peltatum* essential oil might be due to the major compounds, such as myrcene, β-pinene, linalool, α-thujone, α-caryophyllene, β-caryophyllene, germacrene-D, β-cadinene, and caryophyllene oxide. 

Indeed, a few reports have revealed that caryophyllene oxide exhibits anti-inflammatory activity in carrageenan-induced rat and mouse paw oedema at doses of 12.5 and 25 mg/kg [32]; furthermore, Menichini et al. [33] (pp. 670–686) reported that caryophyllene oxide identified in the essential oil of *Teucrium montbretti* inhibited nitric oxide (NO) production. The inhibition of the production of the cytokine interleukin-1β (IL-1β) and interleukin-6 (IL-6) was attributed to the presence of caryophyllene oxide, which was identified in *Cinnamomum osmophloeum* essential oil [34]. The essential oil of *Taxandria fragrans*, which mainly comprises linalool, decreased the production of cytokine (TNF-α) and (IL-6) [35]. Moreover, linalool, one of the major compounds in *C. validus* essential oil, showed anti-inflammatory activity in egg-albumin-induced rat paw oedema [36]. A study performed by Paena et al. [37] (pp. 719–723) revealed that (PGE2) was only inhibited by LPS-stimulated macrophage J774 cells when treated with a high concentration of linalool; the same was observed for cyclooxygenase-2 (COX-2) expression, and linalool was also reported to inhibit the production of NO. Myrcene has been reported to significantly inhibit NO production, thereby demonstrating in vitro anti-inflammatory properties [38]. The anti-inflammatory activity in the essential oil recipe was attributed to β-cadinene, which was found to be the major compound of the oil [39]. α-Caryophyllene and α-thujone were reported to significantly reduce ear oedema in rats [40]. Moreover, α-thujone was able to inhibit the release of (TNF-α) in (LPS)-treated RAW 264.7 cells [41]. Furthermore, mice that were pre-treated with *Pterodon emargina* oil exhibited a reduction of (IL-1) and (TNF-α) levels after the intrapleural injection of carrageenan, and germacreneD was identified as the major compound of the oil [42]. Moreover, the inhibitory effect of *Farfugium japonicum* essential oil in the production of nitric oxide (NO) and (PGE2) in LPS-stimulated RAW 264.7 cells was attributed to β-caryophyllene, the major compound of the oil [41]. Additionally, β-caryophyllene, which was identified in *Salvia* and *Helichrysum* species essential oil, significantly inhibited 5-lipoxyhgenase [43,44].

## 4. Materials and Methods

### 4.1. Plant Material

*P. peltatum* was collected along the roadside on the way to Grahamstown, and the plant’s sample was then taxonomically identified by Mr. T. Dold. The voucher specimen was deposited in the Selmar Schonland Herbarium Grahamstown (GRA) at Rhodes University; the collection number was PR/PL02.

### 4.2. Extraction of Essential Oils

In total, 600 g of fresh and dry (leaf and twig) *P. peltatum* was subjected to a hydro-distillation essential oil isolation method for approximately 5 h using a Clevenger apparatus [45,46]. The extracted essential oils were then collected and stored in airtight amber glass bottles and placed in a refrigerator at 4 °C until the time of analysis.

### 4.3. Analysis of Essential Oils

The chemical composition of the essential oils was analysed using GC and GC-MS instrumentation. The essential oils were analysed using a Bruker 450 Gas Chromatography equipped with a Flame Ionization Detector (FID) and BR HP-5 column (non-polar). The analysis was performed on a fused silica capillary (30 m × 0.25 mm; film thickness 0.25 μm), and helium was used as a carrier gas with a flow rate of 1.0 mL/min. The injector and detector temperature was set to 300 °C. The temperature of the oven was 50–250 °C at 3 °C/min. Next, 0.1 μL of the essential oil was injected manually (split mode, split ratio 1:50). Calculation of the peak area percentage was performed on the basis of the FID signal of the Bruker MS software. 

GC-MS: The analysis of the essential oils was performed on a Bruker 450 Gas Chromatography-300 Mass Spectrometry system. The GC was equipped with a fused capillary HP-5MS, and the capillary column parameter was 30 m × 0.25 mm (film thickness 0.25 μm). Helium was used as a carrier gas at a flow rate of 1.0 mL/min, and the split ratio was 1:50. The oven program started with an initial temperature of 70 °C, and then it was heated to 240 °C at a rate of 5 °C/min; the final temperature was kept at 450 °C, and the run time was 66.67 min. For GC-MS detection, an electron ionization system with an ionization energy of 70 eV was used. The scan time was 78 min, with a scanning range of 35 to 450 amu.

### 4.4. Identification of Essential Oils

The identification of compounds was performed by matching their mass spectra and retention indices with those recorded in the NIST08 library, and by comparing retention indices and mass spectra with values from the literature [15,16,17].

### 4.5. Biological Studies

#### 4.5.1. Experimental Animals

Swiss mice (30–40 g) and Wistar rats (200–300 g) were procured from the South African Vaccine Producers in Johannesburg and kept in an animal holding facility at the WSU Human Biology Department in Mthatha. Ethical clearance for this study was issued by the Walter Sisulu Faculty of Health Sciences ethics committee: ethical clearance certificate no. 003A/2018. The animals were housed under standard conditions, with 6 rats in each cage; the room temperature was maintained at 24 °C, and lighting was either by electric lighting or daylight. The animals had free access to food and water until 8 h before experimentation, when only water was provided to the animals.

#### 4.5.2. Drug Used

Diclofenac was obtained from Reckitt Benckiser Pharmaceutical (PTY) LTD/(EDMS) BPK Elansfontein-South Africa.

#### 4.5.3. Acute Toxicity

The acute toxicity of the oils was evaluated using Swiss mice. Before administration of the essential oils to the animals, the essential oil was emulsified with tween 80 to obtain 5% *v*/*v*. The acute toxicity of the leaf or twig (fresh or dry) essential oil of *P. peltatum* was assessed by using Lorke’s method [47], which consists of 2 phases. The animals were randomly distributed, and the first phase of the test consisted of 3 sub-groups (n = 3) for each dose level of 10, 100, and 1000 mg/kg. The second phase employed 4 subgroups (n = 1) per dose level of 1000, 1600, 2900, and 5000 mg/kg, respectively. Immediately after the treatment, each mouse was placed inside the plexiglass cage and observed for immediate effects up to 30 min, and thereafter for 24 h for lethal effects culminating in death. The LD_50_ of the leaf or twig (fresh or dry) essential oil was estimated as the geometric mean of the lowest dose causing death and the highest dose causing no death according to the formula below:

LD_50_ = √(A × B)

where A is the maximum dose producing 0% death and B is the dose that produces 100% death [47]. From the result of LD_50_, the working doses were determined according to the equation below:

Working doses ≤ ½ (LD_50_)

#### 4.5.4. Analgesic Activity: Tail immersion Test

The analgesic activity was investigated by using the tail immersion method in hot water with the temperature maintained at 55± °C [48]. The experimental animals were divided into 5 groups of 6 rats, as follows:

Group I: treated with 5% tween 80, 10 mg/kg (negative control);
Group II: treated with 100 mg/kg diclofenac (positive group);
Group III: treated with 1 mL of 2% of *P. peltatum* fresh leaf essential oil (P.P.F.L) (100 mg/kg);
Group IV: treated with 1 mL of 2% of *P. peltatum* fresh leaf essential oil (P.P. F.L) (200 mg/kg);
Group V: treated with 1 mL of 2% of *P. peltatum* fresh leaf essential oil (P.P.F. L) (400 mg/kg).

A similar experimental set up was repeated with different sets of animals to assess the analgesic effects of dry leaf (P.P.D.L), fresh twig (P.P.F.T), and dry twig (P.P.D.T) of *P. peltatum* essential oils, respectively. The different essential oil experiments were performed on different days.

The rats were fasted for 12 h with only clean tap water provided for drinking. The animals were pretreated 60 min before tail immersion into hot water. Then, an area of the tail of approximately 3–4 cm was marked and immersed in the water bath thermostatically, which was maintained at 55 °C. The time taken for the rat to flick its tail or withdraw it from the hot water was noted as the pain reaction time or the tail flick latency. The observations were made before and at 1 h, 2 h, 3 h, and 4 h after the administration of the respective treatments.

#### 4.5.5. Anti-Inflammatory Activity: Egg-Albumin-Induced Right Hind Paw Oedema

Wistar rats were used to test for anti-inflammatory activity. Five rats were randomly assigned to one of six groups, as follows:

Group I: treated with 5% tween 80, 10 mg/kg (negative control);
Group II: treated with 100 mg/kg diclofenac (positive group);
Group III: treated with 1 mL of 2% of *P. peltatum* fresh leaf essential oil (P.P.F.L) (100 mg/kg);
Group IV: treated with 1 mL of 2% of *P. peltatum* fresh leaf essential oil (P.P. F.L) (200 mg/kg);
Group V: treated with 1 mL of 2% of *P. peltatum* fresh leaf essential oil (P.P.F.-L) (400 mg/kg).

Separate experiments were similarly conducted with five groups of six animals each to investigate the anti-inflammatory effects of dry leaf (P.P.D.L), fresh twig (P.P.F.T), and dry twig (P.P.D.T) *P. peltatum* essential oils, respectively. The experiments of the different essential oils were performed on different days.

Then, 30 min later, 1 mL of 50% (*v*/*v*) fresh egg albumin was injected into each rat’s right hind paw. Baseline paw measurements were made with Vernier Calipers (Yato) prior to and after 1 h, 2 h, 3 h, and 4 h of egg albumin injection [49].

#### 4.5.6. Statistical Analysis

One-way analysis of variance (ANOVA) with Dunnett’s multiple comparison test was performed using GraphPad Prism to determine the difference between treatment groups. The results were expressed as mean ± standard error of the mean (SEM), and *p* < 0.05 was considered significant.

## 5. Conclusions

This study presents the first comprehensive analysis of the chemical composition of *P. peltatum* essential oils from South Africa. Fifty-three compounds were identified in the essential oil of *P. peltatum*, and the dominating components were camphene, α-terpineol, α-thujone, piperitone, linalool, β-myrcene, germacrene-D, β-caryophyllene, β-cadinene, β-bourbonene, caryophyllene oxide, β-pinene, β-cubenene, α-caryophyllene, α-armophene, phytone, and β-phellandrene. The results showed that *P. peltatum* essential oils from fresh leaf, dry leaf, fresh twig, and dry twig were not toxic, and that they had potent analgesic and anti-inflammatory activity. These results confirmed the great potential of *P. peltatum* essential oils and their use in traditional medicine; therefore, *P. peltatum* essential oils could be used as analgesic and anti-inflammatory agents with high potential in cosmetic and pharmaceutical fields.

## Figures and Tables

**Table 1:** Chemical composition of fresh and dry (leaf and twig) essential oils of *P. peltatum*.

No	Compounds	KI^a^	KI^b^	Fresh Leaf (%)	Dry Leaf (%)	Fresh Twig (%)	Dry Twig (%)	I.M
**1**	*p*-Xylene	883	888	-	1.6	0.8	-	MS^c^, KI^d^
**2**	α-Pinene	939	939	-	1.2	3.0	-	MS^c^, KI^d^
**3**	Camphene	953	954	33.4	3.9	3.6	10.4	MS^c^, KI^d^
**4**	β-Pinene	980	978	4.9	1.4	4.4	6.0	MS^c^, KI^d^
**5**	Myrcene	991	988	5.2	-	10.7	-	MS^c^, KI^d^
**6**	2-Pentylfuran	992	1040	-	2.2	-	-	MS^c^, KI^d^
**7**	2-Carene	1001	1001	-	1.6	1.9	-	MS^c^, KI^d^
**8**	α-Terpinene	1018	1017	-	1.3	1.2	-	MS^c^, KI^d^
**9**	β-Phellandrene	1031	1030	-	-	4.6	-	MS^c^, KI^d^
**10**	trans-β-ocimene	1053	1052	-	2.2	0.9	-	MS^c^, KI^d^
**11**	α-Terpinolene	1088	1079	-	1.4	0.6	-	MS^c^, KI^d^
**12**	Linalool	1098	1095	11.7	1.6	1.9	6.2	MS^c^, KI^d^
**13**	Nonanal	1098	1103	-	2.1	2.1	-	MS^c^, KI^d^
**14**	α-Thujone	1102	1123	-	1.5	-	15.6	MS^c^, KI^d^
**15**	iso-Menthone	1164	1162	-	0.8	-	-	MS^c^, KI^d^
**16**	α-Terpineol	1189	1188	19.1	4.8	5.4	13.1	MS^c^, KI^d^
**17**	Myrtenal	1193	1195	-	2.7	-	-	MS^c^, KI^d^
**18**	Piperitone	1251	1249	12.2	0.9	-	-	MS^c^, KI^d^
**19**	α-Cubebene	1351	1348	-	1.0	2.1	-	MS^c^, KI^d^
**20**	α-Longipinene	1351	1353	-	0.7	1.6	-	MS^c^, KI^d^
**21**	Geranyl acetone	1353	1352	-	3.5	-	-	MS^c^, KI^d^
**22**	Eugenol	1356	1351	-	1.7	-	-	MS^c^, KI^d^
**23**	α-Copaene	1376	1377	-	-	2.4	-	MS^c^, KI^d^
**24**	β-Bourbonene	1384	1388	-	6.2	-	4.2	MS^c^, KI^d^
**25**	β-Cubenene	1390	1387	-	-	5.6	-	MS^c^, KI^d^
**26**	α-Cadinene	1409	1412	-	-	1.7	-	MS^c^, KI^d^
**27**	α-Gurjunene	1409	1410	-	-	0.5	-	MS^c^, KI^d^
**28**	β-Caryophyllene	1418	1419	4.3	1.2	9.5	-	MS^c^, KI^d^
**29**	α-Ionone	1426	1426	-	2.8	2.0	-	MS^c^, KI^d^
**30**	γ-Elemene	1430	1437	-	-	2.8	-	MS^c^, KI^d^
**31**	(Z)-β-farnesene	1443	1457	-	0.7	-	-	MS^c^, KI^d^
**32**	α-Caryophyllene	1454	1478	-	0.8	5.5	-	MS^c^, KI^d^
**33**	γ-Gurjunene	1473	1475	-	1.8	1.4	-	MS^c^, KI^d^
**34**	Germacrene D	1480	1483	-	-	3.7	10.4	MS^c^, KI^d^
**35**	α-Armophene	1485	1484	-	-	1.2	5.2	MS^c^, KI^d^
**36**	α-Selinene	1494	1498	-	0.5	0.6	-	MS^c^, KI^d^
**37**	β-Cadinene	1519	1518	-	-	3.4	6.7	MS^c^, KI^d^
**38**	δ-Cadinene	1524	1522	-	0.9	0.8	-	MS^c^, KI^d^
**39**	α-Calacorene	1548	1545	-	0.8	-	-	MS^c^, KI^d^
**40**	Lauric acid	1568	1570	-	1.5	-	-	MS^c^, KI^d^
**41**	Spathulenol	1576	1578	-	2.6	0.7	-	MS^c^, KI^d^
**42**	Caryophyllene oxide	1581	1582	-	2.6	1.9	6.1	MS^c^, KI^d^
**43**	Cedrol	1596	1616	-	-	1.2	-	MS^c^, KI^d^
**44**	1,2-Epoxide-humulene	1606	1608	-	-	0.9	3.8	MS^c^, KI^d^
**45**	cis-α-Santalol	1678	1678	-	0.5	-	-	MS^c^, KI^d^
**46**	Myristic acid	1720	1746	-	2.0	-	-	MS^c^, KI^d^
**47**	Phytone	1845	1840	-	5.0	0.4	-	MS^c^, KI^d^
**48**	Longifolenaldehyde	1876	1876	-	2.7	-	-	MS^c^, KI^d^
**49**	E,E-Farnesyl acetone	1921	1920	-	1.8	-	-	MS^c^, KI^d^
**50**	Palmitic acid	1984	1984	-	-	1.2	-	MS^c^, KI^d^
**51**	*n*-Eicosane	2000	2000	-	0.6	-	-	MS^c^, KI^d^
**52**	Heneicosane	2100	2100	-	1.0	-	-	MS^c^, KI^d^
**53**	Linoleic acid	2130	2146	-	1.0	-	-	MS^c^, KI^d^

KI^a^: Kovats indices on HP-5MS capillary column. KI^b^: Kovats indices from the literature [15,16,17,18,19,20,21,22,23,24,25,26,27,28,29,30,31,32,33,34,35,36,37,38,39,40,41,42,43,44,45,46,47]. MS^c^: Identification based on mass spectral data. KI^d^: Identification on the basis of NIST11 library and comparison with data from the literature.

**Table 2:** Toxicity assessment of *P. peltatum* essential oils.

Plant Part Used	Dose (mg/kg, p.o.)	Death Patterns after 24 h
	Phase 1 (*n* = 3)	
Fresh leaf, dry leaf, fresh twig, dry twig	10	0/3
Fresh leaf, dry leaf, fresh twig, dry twig	100	0/3
Fresh leaf, dry leaf, fresh twig, dry twig	1000	0/3
	Phase 2 (*n* = 1)	
Fresh leaf, dry leaf, fresh twig, dry twig	1000	0/1
Fresh leaf, dry leaf, fresh twig, dry twig	1600	0/1
Fresh leaf, dry leaf, fresh twig, dry twig	2900	0/1
Fresh leaf, dry leaf, fresh twig, dry twig	5000	0/1
	LD_50_	${\mathrm{LD}}_{50}=\geq 5000$ mg/kg, p.o

## Data Availability

Not applicable.
